# A Brief Synopsis on the Genetics of Alzheimer’s Disease

**DOI:** 10.1007/s40142-018-0155-8

**Published:** 2018-10-30

**Authors:** M. Ilyas Kamboh

**Affiliations:** Department of Human Genetics, Graduate School of Public Health, University of Pittsburgh, Pittsburgh, PA 15261 USA

**Keywords:** Alzheimer’s disease, Genetics, Genes

Alzheimer’s disease (AD) is the most common form of dementia, accounting for 60–80% of all dementia cases [1]. Due to variation in age at onset (AAO), AD is classified as either early onset (AAO < 60 years) or late onset (AAO ≥ 60 years). Approximately 5% of all AD cases are classified as early-onset AD (EOAD) [2], of which10–15% having the family history of disease follow the autosomal dominant inheritance [3] with known mutations in three genes: *APP*, *PSEN1*, and *PSEN2* [4]. Majority of the remaining EOAD cases are sporadic with a complex genetic etiology.

Late-onset AD (LOAD) that comprises about 95% of all AD cases is genetically even more complex with polygenic risk inheritance. LOAD has a substantial genetic component with heritability estimates up to 79% [5]. Until recently, *APOE* was the only established susceptibility gene for LOAD. Substantial progress has been made via large-scale genome-wide association studies (GWAS) as well as meta-analyses of GWAS that have identified more than 30 susceptibility loci for LOAD [6–14], mostly among populations of European ancestry. However, known common polymorphisms at these loci explain 16% of the AD phenotypic variance (or 31% of genetic variance) [15]. Recent application of whole-exome microarray, whole-exome sequencing, whole-genome sequencing, and targeted sequencing has identified rare variants in additional novel LOAD genes [16–24].

The purpose of this brief synopsis is to provide an update on the known number of AD genes and loci that have been independently replicated or meet stringent criteria of genome-wide significance (Table 1). In addition to the three causal genes for autosomal dominant EOAD, to date, 43 susceptibility genes/loci have been identified for LOAD. The *APP* gene for EOAD also harbors a protective variant for LOAD. This list of genes will need to be amended as more genes/loci are discovered and GWAS-implicated loci are refined to identify specific genes. Unlike *APOE* and those LOAD genes where the sequencing approach has revealed rare and coding AD-associated variants, the identity of specific genes driving the associations within most GWAS-implicated loci is not known. Functional follow-up of these genetic loci in in vitro and/or in vivo studies will help to identify the specific genes and their relevance to AD pathology.

**Table 1:** AD-associated genes/loci

Chromosome	Region	Gene/locus	Variant frequency	Variant effect	Reference
Autosomal dominant genes					
1	1q42.13	*PSEN2*	Rare	Risk	[4]
14	14q24.2	*PSEN1*	Rare	Risk	[4]
21	21q21.3	*APP*	Rare	Risk	[4]
LOAD susceptibility genes					
4	4q23	*UNC5C*	Rare	Risk	[21]
6	6p21.1	*TREM2*	Rare	Risk	[17, 23]
7	7q21.2	*AKAP9* ^*1*^	Rare	Risk	[22]
7	7q	AC099552.4*	Rare	Risk	[23]
15	15q21.3	*ADAM10*	Rare/common	Risk/protective	[7, 24]
15	15q26.3	*TM2D3*	Rare	Risk	[20]
16	16q24.1	*PLCG2*	Rare	Protective	[19]
17	17q21.32	*ABI3*	Rare	Risk	[19]
19	19q13.32	*APOE*	Common	Risk/protective	[6–9]
19	19q13.2	*PLD3*	Rare	Risk	[18]
21	21q21.3	*APP*	Rare	Protective	[16]
LOAD susceptibility loci					
1	1q32.2	*CR1*	Common	Risk	[6, 7]
2	2q37.1	*INPP5D/SHIP1*	Common	Risk	[6, 7]
5	5q31.3	*HBEGF/PFDN1* ^2,3^	Common	Risk	[11, 13]
5	5q14.3	*MEF2C/TMEM161B*	Common	Protective	[6]
6	6p12.3	*CD2AP/ADGRF2*	Common	Risk	[6–9]
6	6p21.32	*HLA-DRB1/HLA-DQB1*	Common	Risk	[6, 7]
7	7p12.1	*COBL* ^1^	Rare	Protective	[10]
7	7p14.1	*NME8/EPDR1*	Common	Protective	[6]
7	7q22.1	*NYAP1/PILRA/STAG3*	Common	Protective	[6, 7, 23]
7	7q34-q35	*EPHA1/TAS2R60*	Common	Protective	[6, 7]
8	8p21.2	*PTK2B*	Common	Risk	[6, 7]
8	8p21.2	*CLU/APOJ*	Common/rare	Risk/protective	[6, 7]
10	10p14	*ECHDC3/USP6NL* ^2,3^	Common	Risk	[7, 11, 13]
11	11p11.2	*SPI1/CELF1*	Common	Risk/protective	[6, 7]
11	11q12.1	*MS4A2/MS4A6A*	Common	Protective	[6–9]
11	11q14.2	*PICALM*	Common	Protective	[6, 7]
11	11q24.1	*SORL1*	Common/rare	Risk/protective	[6, 7]
13	13q33.1	*SLC10A2/METTL21EP* ^*1*^	Rare	Protective	[10]
14	14q22.1	*FERMT2*	Common	Risk	[6, 7]
14	14q32.12	*SLC24A4/RIN3*	Common	Protective	[6, 7]
15	15q21.2	*SPPL2A/TRPM7* ^3^	Common	Protective	[13]
15	15q22.31	*TRIP4*	Rare	Risk	[14]
16	16p12.3	*IQCK/DEF8*	Common	Protective	[7]
17	17p13.2	*SCIPM/ZNF594/USP6* ^3^	Common	Protective	[13]
17	17q21.31	*KANSL1/MAPT* ^4^	Common	Protective	[12]
17	17q22	*TSPOAP1/BZRAP1-ASI* ^*2*^	Common	Protective	[11]
17	17q23.3	*ACE*	Rare	Risk	[7]
18	18q12.1	*DSG2/DSG3*	Common	Protective	[6]
19	19p13.3	*ABCA7*	Rare/common	Risk	[6, 7, 9], [23]
19	19p13.3	*NFIC* ^*2*,5^	Common	Protective	[11]
19	19q13.41	*CD33/SIGLECL1*	Common	Protective	[6, 8, 9]
20	20q13.31	*CASS4/GSTF1*	Common	Protective	[6, 7]
21	21q21.3	*ADAMTS1/ADAMTS5*	Common	Protective	[7]

*Long non-coding RNA, ^1^unique in African blacks, ^2^transethnic GWAS, ^3^GWAS by proxy (GWAX), ^4^GWAS among non-APOE*4, ^5^interaction with APOE*4

The ultimate goal of understanding the complete genetic architecture of AD is to discover novel pathways that may converge in the causation of this heterogeneous disease, and to identify drug targets for therapeutic treatment. Manually curated pathways based on a selected set of probable LOAD genes provide some insights about possible disease mechanisms [25], but without including the products (proteins) of all known genuine and yet to be discovered AD genes (both for early and late onset), and those interacting with these genes/proteins, the interpretation of these pathways is incomplete.
